# What is the impact of microbiota on dry eye: a literature review of the gut-eye axis

**DOI:** 10.1186/s12886-024-03526-2

**Published:** 2024-06-19

**Authors:** Jiaping Song, He Dong, Tingting Wang, He Yu, Jian Yu, Shaokang Ma, Xiaohai Song, Qianhui Sun, Yongcheng Xu, Mingkai Liu

**Affiliations:** 1 Department of Clinical Medical Laboratory, The Third People’s Hospital of Dalian, No. 40, Qianshan Road, Ganjingzi District, Dalian City, Liaoning Province 116033 China; 2 Department of Ophthalmology, The Third People’s Hospital of Dalian, Dalian, Liaoning 116033 China; 3 Department of Clinical Laboratory, Dalian University Affiliated Xinhua Hospital, Dalian, Liaoning 116021 China

**Keywords:** Dry eye, Microbiota, Immunity, Gut-eye axis

## Abstract

**Background:**

Dry eye is a chronic and multifactorial ocular surface disease caused by tear film instability or imbalance in the microenvironment of the ocular surface. It can lead to various discomforts such as inflammation of the ocular surface and visual issues. However, the mechanism of dry eye is not clear, which results in dry eye being only relieved but not cured in clinical practice. Finding multiple environmental pathways for dry eye and exploring the pathogenesis of dry eye have become the focus of research. Studies have found that changes in microbiota may be related to the occurrence and development of dry eye disease.

**Methods:**

Entered the keywords “Dry eye”, “Microbiota”, “Bacteria” through PUBMED, summarised the articles that meet the inclusion criteria and then filtered them while the publication time range of the literature was defined in the past 5 years, with a deadline of 2023.A total of 13 clinical and 1 animal-related research articles were screened out and included in the summary.

**Results:**

Study found that different components of bacteria can induce ocular immune responses through different receptors present on the ocular surface, thereby leading to an imbalance in the ocular surface microenvironment. Changes in the ocular surface microbiota and gut microbiota were also found when dry eye syndrome occurs, including changes in diversity, an increase in pro-inflammatory bacteria, and a decrease in short-chain fatty acid-related bacterial genera that produce anti-inflammatory effects. Fecal microbiota transplantation or probiotic intervention can alleviate signs of inflammation on the ocular surface of dry eye animal models.

**Conclusions:**

By summarizing the changes in the ocular surface and intestinal microbiota when dry eye occurs, it is speculated and concluded that the intestine may affect the occurrence of eye diseases such as dry eye through several pathways and mechanisms, such as the occurrence of abnormal immune responses, microbiota metabolites- intervention of short-chain fatty acids, imbalance of pro-inflammatory and anti-inflammatory factors, and release of neurotransmitters, etc. Analyzing the correlation between the intestinal tract and the eyes from the perspective of microbiota can provide a theoretical basis and a new idea for relieving dry eyes in multiple ways in the future.

## Background

Dry eye is a chronic ocular surface disease caused by tear film instability or imbalances in the ocular surface microenvironment and resulted in a range of discomfort symptoms and visual problems. While the exact causes and mechanisms are not fully understood, leading to the situation can only be managed but not cured. The microbiota with many residing on the skin and mucosal surfaces of the host body in a symbiotic relationship. In recent years, research has shown a significant link between microbiota and disease development, making the exploration of this relationship a key area of focus [1]. The unique structure of the ocular surface, constantly exposed to the external environment, results in the development of a stable symbiotic microbiota that plays a crucial role in maintaining the immune balance of the ocular surface. Disrupting microenvironment can lead to various ocular diseases. The gut, extensively studied as a key mucosal site for understanding the human microbiome by the National Institutes of Health (NIH) in the United States, harbors approximately 150 times more microbial genes than human genes [2]. Recent studies demonstrated that changes in gut microbiota can impact the onset of ocular diseases. Investigating the correlation between the microbiome and ocular diseases has emerged as a starting point for examining disease pathways and preventing disease progression. This review aims to delve into the potential mechanisms of the gut-eye axis by analyzing the impact of microbiota on ocular surface immunity and outlining the alterations in the ocular surface and gut microbiota in cases of dry eye, seeking to offer novel insights for the treatment and management of clinical dry eye through a multi-faceted approach.

## Methods

During the search and screening process, the keyword “dry eye” was entered using PubMed. In order to ensure the innovation and timeliness of the article, while screening the literature based on the keywords, the publication time range of the literature was defined in the past 5 years, with a deadline of 2023. In September 2019, a total of 4,701 search terms were obtained. After entering the keyword “Microbiota” at the same time, 37 searches were obtained; after entering the keyword “Bacteria”, 221 searches were obtained. Keywords are not separated using Boolean operators in this process. The corresponding references were reviewed at the same time as inclusion, and screened out 2 articles that met the inclusion criteria (Document 63 and Document 64).

The search results that simultaneously satisfied “Dry eye""Microbiota” and “Dry eye” “Bacteria” were extracted, review articles and research articles using animals as experimental subjects were excluded.A total of 13 articles were screened out, among which dry eye and there are 9 studies on ocular surface microbiota and 4 studies on dry eye and gut microbiota. At the same time of inclusion, taking into account the new concept of fecal microbiota transplantation and further verifying the relationship between the gut and the ocular surface, the team also included an animal-related study based on “fecal microbiota transplantation” in 2016. This work was completed by the cooperation of six members.

## Results

### Characterisation of the ocular surface microbiota

The ocular surface, composed of the cornea and conjunctiva, exhibits fewer microbial gene sequences compared to intestinal mucosa. Low abundance sequences from external sources and pollutants can be considered part of the ocular surface commensal microbiota, have resulted in varied findings in research. Despite advancements in research methods from traditional culture to second-generation sequencing-based assays, there remains no consensus regarding the existence of a core microbial composition on the ocular surface.

Research on microorganisms present on the ocular surface can be traced back to the 1930s. The most frequently identified microorganisms in the conjunctiva of healthy individuals include coagulase-negative staphylococci, P*ropionibacterium spp, Corynebacterium spp*, *Staphylococcus aureus*, *Streptococcus*, as well as Gram-negative bacteria like *Haemophilus species*, *Neisseria species*, and *Pseudomonas species* [3, 4]. With the stability of the genetic code confirmed, sequencing the 16 S ribosomal ribonucleic acid gene (16 S rRNA) has emerged as an improved method for analyzing microbial composition in habitats. These habitats typically consist of five main phyla: *Proteobacteria*, *Actinobacteria*, *Firmicutes*, *Cyanobacteria*, and *Bacteroidetes*. The first three phyla make up more than 87% of the total composition, while *Cyanobacteria* and *Bacteroidetes* were identified as contaminants. In addition to 59 genera, the presence of *Bradyrhizobium*, *Acinetobacter*, *Brevundimonas*, *Aquabacterium*, and *Sphingomonas* was noted, along with genera commonly found in culture methods [5]. In addition to differences in species, there were variations in abundance between the two methods. The dominant bacteria identified through the traditional culture method constituted a significantly lower percentage of the sequencing results. For instance, *Staphylococcus*, which was more prevalent in the former method, accounted for only 4% of the total. This discrepancy could be attributed to the bias of the traditional culture method towards genera that are suitable for growth in the medium. In contrast, the 16 S sequencing method revealed a much more diverse microbial species composition, making it more suitable for analyzing dominant species in the environment. This study had a limited number of subjects for analysis, and a larger sample size would be necessary to validate the findings.

Sampling effort plays a crucial role in the detection of environmentally relevant genera on the ocular surface. Light pressure wiping has been shown to detect genera such as *Rothia*, *Herbaspirillum*, *Leptothrichia*, and *Rhizobium*, while reducing the detection of *Firmicutes* (*Staphylococci*), *Actinobacteria* (*Corynebacterium spp.*), and *Proteobacteria*. On the other hand, strong pressure wiping results in a higher abundance of *Proteobacteria*, *Bradyrhizobium*, *Delftia*, and *Sphingomonas* on the conjunctival epithelium [5]. Deep pressure is recommended over scraping when studying ocular surface microorganisms, as the microbial fraction easily washed away by mucus. Wen et al [6] discovered that older individuals had higher levels of Shannon’s index and increased abundance of *Staphylococcus haemolyticus*, *Micrococcus luteush*, and *E. coli*, while younger individuals had more *Ochrobactrum anthropi*, *Mycoplasma hyorhinis*, and *P. acnes*. Additionally, the elderly group showed higher abundance of conjunctival microbial metabolic pathways related to carbohydrates, fats, nutrients, and amino acids compared to the young group, suggesting that age may have a stronger impact on microbial composition than sex.In a recent study on diabetic dry eye in children and adolescents, similar results to previous findings were observed [5, 7]. The phylum levels of ocular surface microorganisms in normal children and adolescents were mainly composed of *Proteobacteria*, *Firmicutes*, and *Actinobacteria*. However, variations in abundance could indicate a potential correlation with factors such as age and immune status.

Research on the microbiological characterization of the ocular surface has shown a growing trend over the past decade. The results of a comprehensive study indicate that the main phyla present on the ocular surface include *Proteobacteria*, *Actinobacteria*, *Firmicutes*, and *Bacteroidetes*. Sampling technique, environment, age, and gender have been found to influence the microbiological composition of the ocular surface. The definition of the core microbiota remains inconclusive. Despite the various influencing factors, they do not seem to disrupt the normal status of the ocular surface, suggesting the presence of a unique immune system that provides a response to external pathogens while also developing tolerance to commensal microorganisms. Further exploration of the relationship between ocular surface microorganisms and immunity may offer insights into their role in the development of ocular surface diseases.

### Microbiota and ocular immune tolerance

The homeostasis in ocular surface microenvironment is primarily accomplished through mechanical eye movements and the activation of local immunity. Blinking and tear flushing aid in the removal of foreign bodies from the ocular surface.Apart from the actions, the ocular surface houses a natural immune system that regulates host immunity in response to microorganisms. This regulation involves the corneal epithelium, maintenance of corneal avascularity, and interaction with conjunctiva-associated lymphoid tissues and resident immune cells such as secretory IgA (sIgA) and lymphocytes.

The primary antibody produced by Goblet cells in the lacrimal and conjunctival glands is sIgA, which is generated by B cells. These B cells are initially prompted by primitive B cells that travel from the bone marrow to the conjunctiva or lacrimal glands after undergoing class switching. sIgA plays a crucial role in preventing pathogenic bacterial infections by aggregating in the mucin layer, binding to mucin, and also promoting the anti-inflammatory cytokine IL-10, which influences the maturation of dendritic cells [8]. This process ultimately leads to the induction of immune tolerance in the mucous membranes. In research studies, it was observed that ocular surface sIgA levels decreased in conventionally reared mice following oral administration of antibiotics [9]. Conversely, levels of IgA-producing B cells showed a significant increase in germ-free rats after transitioning them to a conventional rearing environment [10]. Additionally, a positive relationship was identified between the diversity of intestinal microbiota and sIgA levels [11]. While there is no direct evidence linking this change to the ocular surface microbiota, it is conceivable that this change could be influenced by alterations in the ocular surface environment or other parts of the host, like the gut. Further research is needed to confirm whether the ocular surface microbiota plays a role in stimulating and transforming B cells. The mechanism by which the microbiota initiates this response remains unclear. Studies have shown that when MyD88 and TRIF are knocked out from the Toll-like receptor activation pathway in the gut, mice experience reduced IgA production [8]. On the other hand, Toll-like receptor stimulation leads to B-cell activating factor promoting IgA class switching through a T-cell-independent pathway, ultimately stimulating IgA production. It is more probable that this antibody production is initiated by recruitment from other mucosal sites, such as the gut, rather than originating from the lacrimal gland.

Various receptors on the ocular surface can respond to different signals that trigger inflammatory pathways.Pathogen-associated molecular pattern receptors located in the ocular epithelium are activated by specific stimuli, initiating innate and specific immune responses through the production of cytokines, chemokine ligands, and the activation of inflammatory pathways like nuclear factor-kB and mitogen-activated protein kinases [4, 12]. TLR4 activation by lipopolysaccharide (LPS) can induce dry eye development by increasing cytokine release in the cornea and conjunctiva [13]. Studies in animal models demonstrated that LPS up-regulates the expression of IL-12a, IL-1β, and IFN-γ in dry eye [13], as well as increasing the production of chemokines associated with Th1 cells, ultimately leading to Th1-related dry eye development. TLR5, found in the conjunctival epithelium, recognizes flagellin proteins from pathogenic bacteria and responds to them [14]. Pathogenic bacteria trigger a response by activating receptors on the ocular surface, while commensal bacteria contribute to mucosal protection by competing with pathogenic bacteria. In vitro studies show that healthy corneal and conjunctival cells do not mount an immune response to ocular surface commensal bacteria like Staphylococcus epidermidis or *Propionibacterium acnes*. Instead, they secrete cytokines like IL-6 and IL-8 in response to pathogens such as *Pseudomonas aeruginosa* [15]. Mice colonised with *Corynebacterium mastitidis* enhance ocular surface immune responses against *Pseudomonas aeruginosa* and *Candida albicans* infections by inducing ocular surface T cells to produce IL-17 [16]. While the exact role of Toll-like receptors (TLR) in the immunopathogenesis of dry eye remains to be fully elucidated, it is plausible to suggest that disturbances in microbiota balance and activation of TLR signaling can trigger immune responses linked to the development of dry eye.

### Dry eye - ocular surface homeostasis imbalance

The healthy ocular surface plays a crucial role in maintaining the eye’s stability. The cornea, lacking blood vessels and lymphatic vessels, is considered an immune-privileged area, limiting the access of immune cells. This helps prevent excessive immune responses on the ocular surface. The balance of angiogenic and anti-angiogenic factors in the corneal epithelium is key to this defense mechanism. Studies have shown that immature antigen-presenting cells at the corneal limbus promote T-lymphocyte tolerance. Anti-inflammatory factors like TGF-β, VIP, and IL-Ra can counteract inflammatory responses by inhibiting the activation of antigen-presenting cells when the ocular surface is compromised.Cells and factors such as regulatory T cells (Treg) and programmed death ligands are expressed on the ocular surface to regulate inflammation and maintain homeostasis. In dry eye conditions, activation of the innate and adaptive immune systems leads to increased infiltration of effector T cells, causing inflammation. NK cells play a crucial role in the early stages of dry eye development by responding rapidly to dryness stress [17], secreting IFN-γ, promoting APC cell maturation, and inducing pathogenic Th17 cell polarization [18], ultimately exacerbating dry eye symptoms. Ocular surface NK cells showed significant correlations with OSDI scores, TBUT, and Schirmer’s test in dry eye patients [19]. However, their percentage remained unaltered, consistent with previous research that found no significant increase in NK cells in the conjunctiva [20]. This discrepancy could potentially be attributed to variations in NK cell status between humans and animals, as well as differing disease states as contributing factors.

Elevated levels of pro-inflammatory factors such as IL-1, IL-6, IFN-γ, and IL-17 have been observed in clinical and animal models of dry eye [18, 21–23]. IL-1 plays a role in stimulating the secretion of chemokines, IL-6, and IL-8 by human corneal epithelial cells [21], as well as inducing the expression of antimicrobial peptides by epithelial cells in the cornea and conjunctiva to bolster ocular surface protection. Correlations have been found between IL-1 levels and corneal fluorescein staining [24]. Additionally, IL-1, in conjunction with TNF-α, facilitates the up-regulation of inter-cellular adhesion molecule on ocular surface epithelium, as well as the expression of co-stimulatory factors (CD80/86), chemokine receptor 7, and MHC-II. This leads to chemotactic leukocyte recruitment and the initiation of early phases of inflammation in the disease.IL-6 concentration in tears showed significant correlations with the severity of ocular surface epithelial lesions, tear film rupture time, Schirmer’s test, tear clearance, keratoepithelioplasty score, and cupped cell density [22]. Additionally, IL-6 was found to inhibit the differentiation of Foxp3 + Treg cells, which, in conjunction with TGF-β, promotes the expression of Th17 cell-associated transcription factors linked to various ocular diseases [25]. Research has shown that IFN-γ-associated Th1 cells and IL-17-associated Th17 cells are distinct cell subpopulations present in the draining lymph nodes of mice in the dry eye model. IL-17 plays a crucial role in disrupting the corneal barrier and is considered a key factor in the progression of dry eye [23]. When mice are subjected to experimental drying stress on the ocular surface, there is an increase in the number of CD4 + T cells in the conjunctival epithelium, along with elevated levels of IL-17 in the cornea, conjunctiva, and tears [23]. IL-17 plays a critical role in promoting inflammation and corneal epithelial barrier dysfunction by upregulating ICAM-1 expression and activating matrix metalloproteinase-9 [23]. Blocking IL-17 has been shown to reduce disease severity and restore Treg function [26]. Additionally, IL-17 contributes to corneal lymphangiogenesis via the VEGFD/C-VEGFR3 signaling pathway, facilitating immune cell transport to the ocular surface and worsening dry eye inflammation. Chemokines such as CCR5 and CXCR3 produced by Th1 cell stimulation recruit more lymphocytes to the ocular surface epithelium of dry-eyed mice, leading to a Th1-type inflammatory response. Increased expression of CCL20 also plays a role in the aggregation of Th17 + cells and the influx of corneal IL-17 + cells involved in Th17 cell homing [23]. Fractalkine/CX3CL1, a potent chemoattractant for CX3CR1 + leukocytes found in normal human tear fluid, is involved in leukocyte activation, transport, and adhesion. In the mouse model of desiccation syndrome, Fractalkine is a key molecule in inducing monocyte infiltration and inflammation [27].

A role for CD4 + T cells in dry eyes has been demonstrated, with clinical and animal models showing increased Th1 and Th17 cells and decreased Treg cells in T cell subsets. Clinical cases of dry eye have also shown increased expression of the IL-23/Th17 axis, leading to higher levels of IL-6, IL-23R, TGF-β2, and the transcription factor RoRγt [23]. In animal models, excessive transfer of CD4 + T cells in dry eye mice exacerbates symptoms in Treg-deficient mice, confirming the suppressive role of Treg cells in dry eye conditions. The dysregulation of Treg cells is linked to various immune disorders, with Th17 exerting an opposing effect on Treg function. Restoring Treg function by blocking IL-17 significantly reduces disease severity [26]. These findings indicate that effector T cells may adapt to dry eye progression by differentiating into specific subpopulations to preserve cellular balance.

Conjunctiva-associated lymphoid tissue and tear drainage-associated lymphoid tissue, along with the lacrimal gland [28], contain abundant plasma cells that produce sIgA)to defend the ocular mucosa against external pathogens. In an experimental model of dry eye, blocking the pathogenic IL-17 associated with dry eye led to reduced formation of germinal centers and decreased transfer of pathogenic B cells [29]. While there is limited research on the role of B cells in dry eye, further studies using animal and clinical models are needed to clarify their mechanisms of action in this condition.

### Dry eye and ocular surface microbiota

The inflammatory nature of dry eye is linked to changes in microbiomics, emphasizing the importance of altered ocular surface microbiota in dry eye development. Recent clinical studies have outlined the variations in ocular surface microbiota in dry eye states, highlighting both similarities and differences compared to normal subjects(Table 1). Some studies have reported a decrease in alpha diversity of ocular surface microbiota in dry eyes [30–32], while others have found no change [33], potentially related to the underlying causes of dry eyes. Meibomian Gland Dysfunction dry eye (MGD) shows no difference in diversity compared to normal eyes or other types of dry eye [32, 33],whereas in the abundance in meibomian gland secretions is lower than healthy [34], which suggests that fewer disease-related microbial species in MGD patients may be more expressed inside the glands. At the same time, it was also found that the study by Dong [33] believed that the diversity of meibomian gland dysfunction dry eye did not change with the severity of the disease, but the study by Jiang [35] found that the detection rate and number of bacterial species in the severe MGD group were both significantly higher than the control group, mild and moderate MGD groups [35]. Another cause of dry eye-Aqueous tear deficiency (ATD) is associated with reduced alpha diversity [31]. Dry eye patients with diabetes exhibit increased diversity [7, 36]. The heightened alpha diversity suggests a state of resistance to inflammation on the ocular surface. In contrast, studies on β diversity consistently show differences between dry eye patients and normal subjects [7, 31, 32, 36].

**Table 1:** Dry eye related with ocular surface microbiota

Author/Date	Subjects	Groups	Comparison group	Diversity changes	Increase	Reduce	Main point
Li [32]	Human	DE: NMGD: Patients suffered from MGD MGD: Patients diagnosed with MGD NDE : Normal subjects	DE vs.NDE	α diversity: NDE> DE β diversity: DE was distinguished from NDE	*Bacteroidia* *Bacteroidetes*	*Pseudomonas_ plecoglossicida* *Pseudomonas_ plecoglossicida* *Pseudomonadaceae* *Pseudomonas* *Gamma-proteobacteria* *Proteobacteria*	1. The most predominant genera were *Pseudomonas* (11.49%), *Acinetobacter* (7.79%), *Bacillus* (7.10%), *Chryseobacterium* (2.84%), and *Corynebacterium* (2.73%) in DE ; 2. The predominant bacterial genera in both MGD and NMGD were *Pseudomonas*, *Bacillus*, *Acinetobacter*,*Corynebacterium*, *Chryseobacterium*,*Pedobacter*, *Sphingomonas*,and *Photobacterium*; 3. NMGD subjects had higher abundances of *Bacteroidetes*.
			MGD vs.NMGD	α diversity: No differences β diversity: No differences	*Bacilli* *Bacillates* *Bacillus pumilus*		
Liang [31]	Human	DED: MGD + ATD + Mixed type Healthy: Healthy individuals	DE vs.Healthy	α diversity: DE< Healthy β diversity: DE> Healthy	*Rothia mucilaginosa* *Malassezia globosa* *Neisseria sbuflava* *Staphylococcus aureus*		1. DED was similar with healthy individuals in the phylum-level composition,*Actinobacteria*,*Firmicutes*, *Proteobacteria*, and *Bacteroidetes* were the major conjunctival microbiota; 2. The dysbiosis in DED is primarily characterized by the depletion of commensal species; 3. The sex-related differences of patients with DED are distinct from that of healthy individuals; 4. The microbial diversity in ATD was lower than mixed dry eye.
Jasmine Andersson [30]	Human	Dryeye: ADDA Control	Dryeye vs.Control	α diversity: Dryeye< Control	*Staphylococcus* *Brevibacterium*	*Pseudomonas* *Corynebacteriu* *Ottowia* *Flavobacterium* *Veillonella* *Rothia* *Microbacterium* *Massilia* *Fusobacterium* *Thermus* *Haemophilus* *Sphingomonas*	1. *Cutibacterium* was the only one genus identified in all patients in the DryEye group; 2. *Enhydrobacter*, *Brevibacterium*, *Staphylococcus*, *Streptococcus*, and *Cutibacterium* may be part of the core ocular surface microbiota; 3. *Chryseobacterium* and *Micrococcus* in the DryEye group were correlated with OSDI or Schirmer’s test.
Dong [33]	Human	MGD: Mild/Moderate/Severe Control	MGD vs.Control	α diversity: No difference β diversity: Significant difference between the severe MGD group and others	*Firmicutes* *Proteobacteria* *Deinococcus-Thermus* *Staphylococcus* *Sphingomonas*	*Actinobacteria* *Corynebacterium*	1. The biomarker phyla were *Proteobacteria* and *Firmicutes* in MGD, and the biomarker genera in the MGD group were *Staphylococcus* and *Sphingomonas*; 2. Meiboscores were positively correlated with the abundance of *Staphylococcus*; 3. There was significant difference in the abundances of *Staphylococcus*, *Corynebacterium*, and *Sphingomonas* between the female patients with and without MGD.
Qi [37]	Human	Dry eye Immdry eye	Dry eye vs.Immdry eye	α diversity: No differences β diversity: Immdry eyes was different from dry eye	*Proteobacteria* *Pelomonas* *Herbaspirillum*	*Actinobacteria* *Firmicutes* *Bacteroidetes* *TM7* *Corynebacterium* *Streptococcus* *Prevotella*	1. The phyla biomaker was *Proteobacteria* in Dry eye and *Actinobacteria*, *Firmicutes*,and *Bacteroidetes* in Immdry eye; 2. The genus biomaker was *Pelomonas* in Dry eye and *Corynebacterium* in Immdry eye; 3. *Herbaspirillum* and *Pelomonas* was negatively correlated with Meibomian gland dropout score and *Pseudomonas* was negatively correlated with tear meniscus height in Dry eye group.
Zhang [36]	Human	DED: Pure dry eye DM + DED: DM patients with dry eye Control	DED vs.DM + DED	α diversity: Lower in DED β diversity: DM + DED was different from DED	*Proteobacteria* *Corynebacterium*	*Actinobacteria* *Bacteroidetes* *Ochrobactrum* *Bacillus* *Cupriavidus* *Lactococcus* *Unclassified Clostridiale* *Lactobacillus*	1. DM + DED has unique core members :*unclassified Ruminococcaceae*, *Bacteroides*,*unclassified Peptostreptococcaceae*, *unclassified Barnesiellaceae*; 2. *Pseudomonas* was the only core members in DED.
			DED vs.Control	α diversity: No differences β diversity: DED was different from control	*Bacteroidetes* *Methylobacterium* *unclassified Rhizobiales* *Amycolatopsis* *unclassified Xanthomonadaceae*		
Chen [7]	Human	DM-DE: Diabetes children and adolescents with dry eye NDM: Healthy controls	DM-DE vs.NDM	α diversity: DM-DE> NDM β diversity: DM-DE> NDM	*Firmicutes* *Bacteroidetes * *Tenericutes* *Acidobacteira* *Acinetobacter* *Lactococcus* *Bacteroides* *Staphylococcus*	*Pseudomonas* *Paenibacillus* *Rhodococcus*	1. *Lactococcus*, *Bacteroides*, *Acinetobacter*, *Clostridium*, *Lactobacillus*, and *Streptococcus* are the unique flora of the DM-DE group.
Fuxin Zhao [34]	Human	MGD: Patients with MGD HC : Absence of any dry eye symptoms; Absence of any symptomology that are criteria for diagnosing MGD; Corneal fluorescein staining was negative	MGD vs.HC	Meibomian gland secretions: Chao1:MGD< HC Shannon and Simpson : No significant differences	*Meibomian gland secretions: * *Rubrobacter* *Novibacillus* *Campylobacter* *Geobacillus* *Sphingomonas* *Corynebacterium* *Sphingobium* *Pedobacter* *Fictibacillus* *Enterococcus* *Sphingobium sp.SYK-6*	*Proteobacteria*	1. *Geobacillus*,*Sphingomonas*,*Sphingobium*, *Pedobacter*,*Fictibacillus*,and *Enterococcus* was specific to the microbiome of the Meibomian gland secretions ; 2. The detection rate of pathogens in the secretions of MGD patients is lower than that of HC; 3. The secretions of MGD patients showed increased expression levels of genes related to carbohydrate metabolism, fatty acid elongation, biosynthesis and degradation, glyceride metabolism.
Xiaodan Jiang [35]	Human	MGD: Mild/Moderate/Severe Control	MGD vs.Control	Meibomian gland secretions: MGD(Severe)> Control	*Corynebacterium macginleyi* *Staphylococcus*		1. The detection rate of bacteria in Meibomian gland secretions of MGD patients was significantly higher than that in the conjunctival sac (CS), the bacterial composition of MG is more complex than that of CS; 2. *Corynebacterium macginleyi* was only detected in the severe MGD group, with a detection rate as high as 26.3%.

Li et al. [32] found that the dominant ocular surface bacteria in dry eye, *Corynebacterium* and *Staphlococci epidermidis*, were altered to include *Pseudomonas*, *Acinetobacter*, *Bacillus*, *Chryseobacterium*, and *Corynebacterium*, potentially impacting ocular surface immunity and IgA production. Dry eyes are typically categorized as lipid-abnormal or aqueous-deficient based on tear composition. *Bacilli* abundance, associated with uveitis and ocular surface infections, was higher in lipid-abnormal dry eyes like MGD [38]. *Staphylococcus* and *Sphingomonas* were identified as signature genera of MGD, with enrichment of *Acinetobacter sp. WCHA45*, *Deinococcus sp. NW-56*, and *Staphylococcus aureus* [31]. *Corynebacterium* was more prevalent in mild MGD [33]. *Sphingomonas* has been linked to endophthalmitis development [39], while *Staphylococcus* has been linked to post-cataract surgery complications like bacterial keratitis, conjunctivitis, and endophthalmitis. This association may be attributed to the notably higher lipase content of *Staphylococcus* found on the ocular surface. This high lipase content can potentially impact the lipid layer composition in individuals with MGD, worsening tear film instability and inflammation on the ocular surface [40]. *Corynebacterium* stimulates T cells to produce IL-17, which serves a protective function [16]. A decrease in *Corynebacterium* levels has been linked to the onset of fungal keratitis [29]. The continuous cycle of bacterial-induced blepharitis further supports the worsening of dry eye inflammation over time. However, it is important to note that in this study, the sampling site included the eyelids categorized as skin, raising questions about whether the high prevalence of S*taphylococci* can be solely attributed to the microbiota of the ocular surface conjunctiva. Interestingly another finding was obtained in the meibomian gland secretions (meibum) of MGD. The abundance of *Campylobacter coli*, *Campylobacter jejuni* and *Enterococcus faecium* was significantly increased in meibum, while it was almost not detected in healthy controls [34]. This special microbiota also exhibited a significant relationship with carbohydrate metabolism, fatty acid elongation, biosynthesis, and degradation. Changes in gene expression levels related to, glyceride metabolism and other related gene expression levels can enable immune evasion through the Type IV secretion system [34]. There are not many analyzes of meibomian glands, and some studies believe that the identification results of meibomian gland secretions may be affected by the deep and superficial layers, and as the disease deepens, its composition becomes increasingly complex [35], which further illustrates the disease is responsible for the etiology of MGD. By identifying the unique functions and metabolic pathways of the microbial community in MGD patients, it can provide another way to explore the pathogenesis of MGD, and also provide a potential target for the development of new treatment strategies.Various studies have reported different findings regarding anterior blepharitis associated with ATD. Liang et al. [31] identified elevated levels of *Janibacter melonis* in anterior blepharitis, while another study found *Enhydrobacter* and *Brevibacterium* to be marker genera of the condition [30]. Given that the subgroup of patients with anterior blepharitis in this study included individuals with graft-versus-host disease, it is postulated that the presence of this immune disorder may influence the identification of dry eye markers, warranting further validation. Dry eye with systemic factors is characterized by involvement of multiple ocular sites, greater damage to ocular surface cells, and challenges in treatment. Moreover, compared to simple dry eye, ocular surfaces of individuals with autoimmune diseases exhibit higher levels of *Corynebacterium*, *Staphylococcus*, and *Prevotella*, along with decreased levels of *Pelomonas* and *Herbaspirillum* [37]. The unique characteristics of *Corynebacterium* cell wall can impact macrophage function. In the study, correlations were identified between *Herbaspirillum* and *Pelomonas* with blepharoplakia loss score, time to first tear film break-up (FTBUT), and lipid layer score. Furthermore, the combination of *Corynebacterium* and *Pelomonas* is believed to be able to differentiate markers of immune dry eye from simple dry eye. The development of immune dry eye is also associated with increased expression of signaling pathways related to cell growth and apoptosis. Dry eye patients with diabetes mellitus exhibit a reduction in ocular surface antimicrobial substances, leading to greater diversity and abundance of their ocular surface microbiota [36]. Diabetic patients may experience corneal nerve damage, resulting in increased tear film instability and decreased TBUT [36]. A study [7]conducted in Shanghai focused on characterizing the ocular surface microbiota of diabetic dry eyes in children and adolescents. The study identified core genera such as *Pseudomonas*, *Paenibacillus*, *Lactococcus*, *Bacteroidetes*, *Acinetobacter*, and *Rhodococcus*, along with a high abundance of *Staphylococcus* and *Staphylococcus aureus*.*Staphylococcus aureus* could impact lipid secretion from the lid glands, contributing to tear film instability. This indicates that the pathogenesis of diabetic dry eye may share similarities with severe MGD [33]. *Lactococcus*, commonly utilized as a probiotic, was found to be more prevalent in children with diabetic dry eyes, potentially linked to its role in regulating NF-KB and STAT-3 signalling pathways [41]. The variations in properties displayed by the ocular surface microbiota in dry eyes highlight the intricate nature of this condition, emphasizing the necessity for a thorough and multifaceted investigation into the connection between ocular surface microbiota and dry eyes.

### Dry eye and gut microbiota

The interaction between the gut microbiome and the immune system is crucial for maintaining intestinal balance and preventing disease. Commensal microorganisms in the gut help protect the host by inhibiting pathogen growth, breaking down indigestible polysaccharides to produce short-chain fatty acids (SCFAs) like butyric acid [42], which have strong immunomodulatory effects. These SCFAs also enhance the intestinal mucosal barrier, defending against pathogens and exhibiting anti-inflammatory properties. Disturbance in the balance of symbiotic bacterial composition can lead to a variety of immune diseases. LPS in the gut trigger local inflammation, allowing immune cells to travel to distant areas like the retina [43]. This implies that alterations in gut commensal bacteria can impact the immune status of the ocular surface. Disruption of intestinal homeostasis can result in pathogenic microorganisms breaching the intestinal mucosal barrier, leading to the release of inflammatory factors and activation of T and B lymphocytes, culminating in disease development. The inflammatory byproducts are then carried by lymphatic vessels to distant tissues, including the ocular surface. Recent studies have gradually confirmed the connection between imbalanced intestinal microbiota and ocular diseases (Table 2).

**Table 2:** Dry eye related with gut microbiota

Author/Date	Subjects	Groups	Comparison group	Diversity changes	Increase	Reduce	Main point
Roberto Mendez [44]	Human	SDE: Patients with Sjogren’s dry eye NDE: Patients with dry eye Control: No dry eye	SDE + NDE vs.Control	Shannon’s diversity Index: No differences Faith’s phylogenetic diversity: SDE + NDE> Control	*Proteobacteria * *Actinobacteria * *Bacteroidetes * *Actinomycetaceae* *Eggerthellaceae * *Lactobacillaceae* *Akkermanciaceae* *Coriobacteriaceae* *Eubacteriaceae * *Megasphaera* *Parabacteroides* *Prevotella*	*Firmicutes* *Faecalibacterium * *Viellonella Ruminococcaceae * *Lachnospiraceae* *Clostridiales* *Bacteroides*	1. The changes in phyla and genera compared to controls were not to be driven by age or by the presence of a comorbid autoimmune disease.
Jayoon Moon [45]	Human	SS: Subjects with Sjogren’s syndrome DES: Subjects with environmental dry eye Controls: Healthy	SS vs.Control	α diversity: No differences β diversity: Significant differences	*Bacteriodetes * *Veillonella* *Eubacterium hallii*	*Firmicutes/Bacteroidetes* *Actinobacteria* *Clostridia * *Bifidobacterium* *Blautia* *Dorea* *Agathobacter*	1. NEI score had positive relation with *Bacteroidetes* and negative relation with *Bifidobacterium*; 2. Tear secretion was significant positive relation with *Actinobacteria* and *Bifidobacteria*; 3. TBUT have negative relation with *Bacteroidetes* and strong positive relation with *Actinobacteria* and *Bifidobacteria*.
			DES vs.Control	α diversity: No differences β diversity: No differences	*Veillonella*	*Subdoligranulum*	
Laura Schaefer [46]	Human	SS: Sjogren’s syndrome patients Dry eye: Dry eye patients Healthy: Healthy controls	SS vs.Healthy	Shannon diversity index: SS< Healthy Chao richness: No differences β diversity: Significant separation	*Bifidobacterium bifidum*	*Bacteroides caecimuris* *Mediterranea masilliensis* *Bacteroides coprophilus* *Clostridium_sp_7_3_54FAA*	1. More severe disease was positively correlated with decreased *Bacteroides caecimuris*, *Mediterranea masilliensis*,*Bacteroides coprophilus* and *Clostridium_sp_7_3_54FAA* and increased *Bifidobacterium bifidum*; 2. Mice colonized with SS patient fecal microbiota showed significantly decreased levels of CD45^+^CD4^+^ FOXP3^+^ cells in CLN tissue compared with mice colonized with healthy microbiota.
			Dry eye vs.Healthy	β diversity: No differences			
Cintia S. de Paiva [47]	Mice	NS: Healthy control DS10:Desiccating stress for 10 days Baseline: Healthy control ABX + DS10:Antibiotic cocktail + desiccating stress for 10 days	DS10 vs.NS	Shannon diversity Index: DS10> NS β diversity: Significant separation	*Proteobacteria*		1. Desiccating stress causes a significant increase in *Proteobacteria*; 2. Oral antibiotic treatment along with DS results in extreme changes in the gut microbiota, caused by in a reduction of commensal bacteria and increase in *Proteobacteria* that was associated with a more severe ocular phenotype.
			ABX + DS10 vs.Baseline	Shannon diversity Index: ABX + DS10< Baseline β diversity: Significant separation	*Proteobacteria* *Enterobacter* *Parasutterella* *Escherichia/Shigella* *Pseudomonas* *Staphylococcus*	*Bacteroidetes* *Firmicutes* *Blautia* *Alistipes* *Lactobacillus* *Allobaculum* *Bacteroides* *Desulfovibrio* *Intestinimonas* *Clostridium*	
ARJUN WATANE [48]	Human	Donor: Healthy human Baseline: Recippients with Sjögren syndrome	Recipient vs.Donor	α diversity: Recipient> Donor	*Actinobacteria * *Bacteroidetes* *Cyanobacteria* *Firmicutes* *Proteobacteria * *Verrucomicrobia * *Parabacteroides goldsteinii * *Alistipes* *Streptococcus * *Blautia*	*Euryarchaeota * *Fusobacteria * *Streptococcus thermophillus* *Faecalibacterium * *Prevotella * *Ruminococcus*	1. There were no significant relationships between diversity indices and DE metrics; 2. DE symptoms (via DEQ5 and OSDI) were negatively correlated with the diversity index, indicating that higher symptom severity was associated with lower diversity; 3. Significant positive correlations were found between effector T cells and regulatory T cells, with both T cell populations were positively correlating with DE symptom severity, while corneal staining was positively correlated with effector T cells and negatively correlated with regulatory T cells.

In a 2020 study examining changes in the gut microbiome of patients with Sjögren’s syndrome-associated dry eye (SS-Dry eye) [44], similar alterations to those observed in other immune disorders [49, 50] were identified in SS-Dry eye. Includeding a decrease in the abundance of the butyrate-producing bacterium *Faecalibacterium*, as well as reduced levels of Treg-inducing *Clostridiales* and *Bacteroides*, which play a role in suppressing the inflammatory response in Th17 cells [47]. Intestinal commensal bacteria play a role in achieving mucosal immune tolerance by balancing Th17 and Treg cells. Changes in gut microbiota in SS-Dry eye patients suggest a link between gut microbiota and the robust immune response at the ocular surface. This raises the question: could this change be influenced by autoimmune antibody factors in SS subjects? A comparative study on environmental factors and SS-associated dry eye revealed both similarities and differences in the results of the two causative groups of dry eye [45]. The pathogenesis of environmental dry eye differs from that of SS dry eye [51], with the former showing intermediate changes in gut microbiota between SS-Dry eye and healthy individuals. Both groups exhibited an increase in *Veillonella*, while environmental dry eye displayed a notable decrease in *Subdoligranulum*. Additionally, SS-Dry eye showed a decrease in the *Firmicutes*/*Bacteroidetes* ratio and a decrease in *Bifidobacterium*, indicating potential intestinal dysbiosis and the initiation of chronic inflammation [52]. A previous study [47]also observed a reduction in the butyrate-producing bacterium *Eubacterium hallii* in SS-Dry eye. Butyrate, known for its anti-inflammatory properties and maintaining the colonic epithelial barrier, may suggest an imbalance in butyrate-associated immunomodulatory mechanisms and intestinal barrier function. Conversely, the β-diversity of environmental dry eye does not show significant differences compared to healthy [45, 46], with a composition that appears more akin to normal. Notably, high levels of *Bifidobacterium bifidum* was identified in SS through metagenome [46]. *Bifidobacterium* is commonly used as a probiotic in animal studies to reduce inflammation in mouse models of SS [41]. However, in the current study, it may be implicated in the ocular pathology of SS. The findings related to *Alistipes* in this study are contradictory to previous research [45]. These variability in the functions of the same genera suggests that further functional studies on commensal bacteria are essential to explore the role of specific strains in disease development. More research is needed to determine if there is a causal relationship between certain strains and ocular disease. To further investigate the potential role of gut-microbiota in influencing ocular phenotype, researchers conducted transplanted with humanised faecal bacteria obtained from individuals with dry eye to germ-free mice. The ocular-cervical lymph nodes of the humanised mice exhibited low levels of CD4^+^CD45^+^Foxp3^+^Treg and more severe signs of corneal destruction. Additionally, a notable decrease in CD4^+^ Treg was observed in the cervical lymph nodes and spleens of the offspring of the colonised mice.Treg levels were found significantly decreased in the cervical lymph nodes and spleens of offspring from colonised mice, indicating that the development of Treg cells regulated by intestinal microbiota may impact subsequent generations through vertical transmission. This suggests a potential genetic component in the development of dry eye in children. Sterile mice colonised with humanised faeces from dry eye patients showed ocular surface symptoms. Additionally, an animal study revealed that altering gut microbiota before exposure to dry-stress resulted in significant changes in gut microbiota, leading to increased global cell loss and disruption of the corneal barrier, potentially linked to a reduction in commensal bacteria and an increase in pathogenic bacteria [47]. Intestinal interventions may serve as a potential avenue for addressing ocular surface inflammation. The efficacy of fecal transplants in treating intestinal conditions like ulcerative colitis, suggesting a potential therapeutic option for immune disorders linked to intestinal dysbiosis. A study conducted by ARJUN WATANE et al. [48] highlighted the promising role of fecal transplants in alleviating symptoms of immune-mediated dry eye. However, further research is needed to address key aspects such as measurement control, identification of optimal donor microbiota composition, and potential impact of varying dietary habits and living environments of donors and recipients.

Limited research exists on the relationship between gut microbiota and dry eye, factors such as disease duration and severity may impact gut microbiota changes. Sjögren’s syndrome (SS) is typically diagnosed late making it challenging to identify correlations between specific antibodies like SSA/SSB and gut microbiota alterations. Another point, current methods rely on 16 S rRNA, potentially missing subtle changes. Establishing a deeper connection between gut and eye requires extensive animal experiments and histological studies.

### Gut microbiota and other eye diseases

#### Uveitis

Uveitis is a prevalent eye disease and a major cause of blindness. Abnormal autoimmune responses and inflammation are playing significant roles in its development. Similar to AMD and SS-Dry eye [47, 53], both patients and animal models of uveitis show a decrease in the diversity and number of intestinal microbiota, making it easier for pathogenic bacteria to colonize [47, 54]. A reduction in beneficial butyrate-producing and anti-inflammatory bacteria such as *Faecalibacterium*, *Bacteroides*, *Lachnospira*, *Ruminococcus*, *Lachnospiraceae*, and *Ruminococcaceae families.*An increasing in the genera *Prevotella*, *Lactobacilli*, *Anaeroplasma*, *Parabacteroides*, and *Clostridium* was also observed in the intestinal tract of mice with Experimental Autoimmune Uveitis [53, 55]. The molecular basis of how altered gut microbiota affects uveitis remains unclear. It is hypothesized that disruption of the blood-retinal barrier by autoreactive T cells targeting retinal antigens, possibly induced by commensal bacteria from the gut [56]. Uveitis is an inflammatory bowel disease, accounting for approximately 4-6% of cases also support a potential connection between gut and eyes [57], where microbial antigens from the intestines could trigger ocular inflammation by promoting the development of auto-reactive Th17 cells and other T-helper cells.

#### Age-related macular disease (AMD)

AMD is characterized by dysfunction of retinal pigment epithelium cells and loss of photoreceptor cells. Various factors, including diet influence the development of AMD.Studies have shown a connection between gut microbiota and neovascular AMD in both animal and clinical research. Dietary habits can influence the composition of gut microbiota, potentially impacting the progression of AMD [53, 58]. A high glycemic index diet is a significant risk factor for the development and progression of AMD in individuals without diabetes. This type of diet is linked to specific changes including a decrease and loss of RPE pigmentation, build-up of lipofuscin, and deterioration of photoreceptor cells in animal studies. High-fat diet can worsen choroidal neovascularization, increase intestinal permeability, and promote the production of inflammatory molecules in mouse model by enhancing the presence of *Firmicutes*. Research has also identified an increase in pro-inflammatory bacteria *Anaerotruncus* and *Oscillibacter*, which contribute to intestinal permeability, in the intestines. Moreover, higher levels of *Ruminococcus torques* and *Eubacterium ventriosum*, associated with a high-fat diet were also observed. Reductions in glutamate, the primary excitatory neurotransmitter in the retina, have been linked to impairments in retinal neurotransmission, while elevated levels of arginine have been correlated with progressive choroidal retinal atrophy.

#### Bacterial keratitis (BK)/ fungal keratitis (FK)

Keratitis is an inflammatory disease of the eye, studies have found that the diversity of ocular surface flora changes when bacterial keratitis occurs [59, 60], and intestinal commensal bacteria can affect the susceptibility to ocular keratitis by affecting sIgA levels [61]. Animal models have shown that gut microbiota can provide protection against *Pseudomonas aeruginosa*-induced keratitis by regulating mature neutrophils. An imbalance in gut microbiota can increase susceptibility to ocular keratitis, leading to higher bacterial load in the cornea and increased production of inflammatory factors [61]. Furthermore, in BK, there is a decrease in *Firmicutes* and an increase in pro-inflammatory bacteria such as *Prevotella copri*, *Bilophila*, pathogenic *Enterococcus*, *Bacteroides* (*B. fragilis*), and *CF231* genera, along with the presence of gastroenteritis-inducing *Dysgonomonas* in immunocompromised patients. This is coupled with a decrease in the anti-inflammatory bacterium *Blautia* [62].

FK is an infectious corneal disease associated with a high risk of blindness, caused by pathogenic fungi. Similar to other ocular diseases, patients with FK show a reduction in the diversity of intestinal microbiota, and decrease in genera such as *Faecalibacterium prausnitzii*, *Megasphaera, Mitsuokella multacida*, and *Lachnospira*. Conversely, there is an increase in pro-inflammatory bacteria like *Enterobacteriaceae* and pathogenic bacteria such as *Shigella*, *Treponema*, and *Bacteroides fragilis*. Notably, *Shigella* has also been associated with decreased butyrate production [63, 64].

Studies are increasingly revealing the impact of microbiota and their byproducts on ocular inflammation and immunity. These findings support the concept of an gut-eye axis, shedding light on how gut microbiota influences ocular surface diseases.

#### The gut-eye axis hypothesis

The imbalance of the ocular surface microenvironment, inflammation, imbalance of the Th17/Treg, abnormal activation of the immune system are key factors in the development of dry eye [26]. Considering immune balance and metabolite production induced by gut microbiota, it is reasonable to suggest that gut microbiota may influence eyes by affecting host immunity, creating a potential gut-eye axis. This concept was supported in a study using an animal model which interventions targeting the gut, such as fecal transplants containing a mixture of probiotics and improved dry eye symptoms [41]. These interventions targeted the gut were linked to improved ocular surface inflammation and signs, providing further evidence of the role of gut microbiota in ocular diseases and suggesting the existence of an gut-eye axis.

The potential mechanisms of the gut-eye axis can be summarized as follows: 1. Myeloid cells acting as triggers. Intestinal commensal bacteria can development and activation of macrophages originating. Myeloid cells like CD103^+^CXCR1^+^ dendritic cells or macrophages may migrate from the gut to the ocular surface and leading to the activation of T-cells which then travel to the eye through lymphatic drainage fluid to exert their effects. 2. An imbalance between pro- and anti-inflammatory cells.The decrease in Tregs can lead to an increase in gut-derived helper T cells Th1 and Th17 cells, which migrate to the ocular surface and lacrimal glands and then produce cytokines, causing damage to the ocular surface. 3. Disrupt the production of SCFA. SCFAs play a significant role in modulating both the proximal and distal immune system, with their anti-inflammatory properties extending from the gastrointestinal tract to the ocular surface. A decrease in SCFA levels can compromise the anti-inflammatory functions of macrophages.A decrease in the abundance of *Faecalibacterium*, a key butyrate-producing genus has been observed in individuals with SS-dry eye and FK [44, 47, 63]. 4. Molecular mimetic modeling suggests that autoreactive T cell-mediated autoimmune responses may arise due to the cross-reactivity between microbial peptides and self-antigens. Pathogenic Th17 cells have the ability to migrate from the gut, to the ocular surface contributing the autoimmune diseases through this cross-reactivity mechanism. The generation of these pathogenic Th17 cells may be modulated by factors such as IL-23 and dietary components [65]. 5. The T-cell threshold model suggests that Th17 cells, which are activated by gut microbes, may travel to target organs through draining lymph nodes. This migration can decrease the activation threshold for autoreactive T cells, including Teff cells. 6. The neuropeptide cycle hypothesis suggests that neuropeptide Y, substance P, and vasoactive intestinal peptide from the gut are crucial in regulating tear secretion [66]. Given the abundance of nerve distributions in the eye, exploring how this gut-derived neuropeptide cycle impacts tear secretion in the lacrimal glands could offer further insights into the intricate gut-eye axis.

## Discussion

The intestine is a complex organ containing trillions of microbial inhabitants that significantly contribute to digestion as well as the development and maintenance of the immune system. The overall health of the host is closely linked to the balance or imbalance of these intestinal microorganisms. Given its distinct immune and physiological properties, the intestinal microbiota has become a major focus of research for exploring potential mechanisms involved in the onset and progression of various diseases. One emerging area of interest is the connection between gut microbiota and eye diseases, known as the ‘gut-eye axis.’ Studies have indicated that disturbances in intestinal microbiota are related to multiple eye conditions such as AMD, uveitis, and corneal inflammation. Notable disparities have been documented in the gut and ocular surface microbiota composition among individuals with eye disease and those who are healthy, potentially impacting the development and progression of such conditions. Various sequencing techniques can yield different types of bacteria at the genus or species level. Presently, most studies on the connection between ocular surface diseases and microbiota depend on 16s rRNA sequencing, concentrating on alterations in diversity and structure. Metagenomics is applied to examine the ocular surface, where bacterial presence is limited. This method enables prompt identification and response to newly detected pathogens. The detailed resolution of metagenomics assists in distinguishing between beneficial and potentially harmful bacteria, including fungi and viruses, as evidenced in bacterial infections such as keratitis. Certain pathogens like Cutibacterium acnes, Staphylococcus aureus, Moraxella lacunata, Pseudomonas alcaligenes, and HSV Simplex virus type 2 have been recognized [67], emphasizing the potential for personalized treatment strategies based on the individual’s microbiome profile [68, 69]. While metagenomics shows promise for investigating microbiome-disease connections, its substantial initial expense poses a challenge to clinical investigations. Nonetheless, simply identifying microbial species does not fully elucidate the microbiota’s impact on dry eye pathogenesis. Further research into community relationships is crucial for uncovering the underlying mechanisms and pathways.Current studies primarily focus on the complex interaction between gut microbiota and the host’s immune system. For example, metabolites produced by gut microbiota, such as short-chain fatty acids, can migrate to the eyes via the blood circulation and boost the generation of ocular Tregs. These Tregs aid in suppressing exaggerated immune responses, preserving immune tolerance, and regulating the immune equilibrium and inflammatory reaction in the eye. This investigation also provides new potential treatment targets for eye ailments, including utilizing probiotics, prebiotics, and antibiotics. Adjusting the gut microbiota composition, like through fecal microbial transplantation, may potentially confer benefits for eye diseases. While this approach has displayed encouraging outcomes in animal models, difficulties emerge due to the complexity of human dietary habits compared to the relatively simplistic animal diet. Variations in dietary choices and behaviors among individuals can impact the efficacy of probiotics and prebiotics, making research interpretation more intricate. In addition to the variability in efficacy caused by factors such as diet, gender, and geographical location, there are also different ‘Intestinal Type’ and ‘Eye Community State Types’ among individuals. It is important to explore these variations separately and develop personalized plans, which may aid in improving the diagnosis of DED and achieving the best treatment outcomes [70, 71]. The connection between changes in gut microbiota and shifts in ocular surface microbiota, as observed in dry eye syndrome, remains uncertain. Future studies should concentrate on unraveling the mechanisms that connect gut microbiota to eye disorders, pinpointing particular bacterial strains and metabolites linked to eye wellness, and performing animal and clinical trials to confirm their efficiency and safety. Additionally, exploring the influence of age, sex, and other variables on the correlation between gut microbiota and eye diseases is crucial.

## Conclusions

Recent studies have found a notable link between gut microbiota and eye conditions, referred to as the ‘gut-eye axis’. Disruption in gut microbiota can affect overall and ocular immune responses via different routes, potentially resulting in eye disorders. Treatments like fecal microbiota transplantation targeting gut microbiota regulation could have a positive effect on eye conditions. Although this field of research is still in the investigative and theoretical stage, it shows potential in unveiling the precise connections between gut microbiota and eye conditions, providing fresh possibilities for preventing and managing such conditions.

## Data Availability

No datasets were generated or analysed during the current study.

## Abbreviations

NIH: National Institutes of Health
16S rRNA: 16 S ribosomal ribonucleic acid gene
sIgA: Secretory IgA
TLR: Toll-like receptors
TLR4: Toll-like receptors 4
TLR5: Toll-like receptors 5
LPS: Lipopolysaccharide
Treg: Regulatory T cells
MGD: Meibomian Gland Dysfunction dry eye
ATD: Aqueous tear deficiency
FTBUT: First tear film break-up
SCFAs: Short-chain fatty acids
SS-Dry eye: Sjögren’s syndrome-associated dry eye
SS: Sjögren’s syndrome
AMD: Age-Related Macular Disease
BK: Bacterial Keratitis
FK: Fungal Keratitis
